# Obstetric Inaugural Dissertation on Prolapsus of the Umbilical Cord

**Published:** 1843-04

**Authors:** 


### ART. II.

*Dissertatio Obstetrica Inauguralis de Prolapsu Funiculi Umbilicalis.* Auctore JOH. CHRIST. SAXTORPH.—*Havniæ*, 1842.

*Obstetric Inaugural Dissertation on Prolapsus of the Umbilical Cord.* By J. C. SAXTORPH.—*Copenhagen*, 1842. Two Parts. 8vo, pp. 64, 68.

IT has long been a matter of regret to us that the medical theses of this country have been rather looked upon as a matter of mere form connected with the process of graduation at a university, than as a means for testing the knowledge and abilities of the student, still less as the early attempt in authorship of a young man who is thus giving his first earnest of what he *can* and of what he one day *will* do. In the medical universities of the continent this is not generally the case; a large number of inaugural dissertations are yearly put forth in the German, Dutch, Danish, and Swedish universities, which bear the marks of much learning and inquiry, and sometimes so much original research as to make them valuable additions to our literature. We could enumerate very many such not only in former times, but in later years; in many instances they have been the commencement of still more extended investigation and discovery, and have paved the way to the future distinction and celebrity of the young author. To the medical professors of the continental universities is due much credit and praise for the exertions which they have made to rouse into activity the talents of their pupils in their first attempt at authorship, and by a little judicious interest and assistance to encourage them in their undertaking; hence it is customary for a professor to reserve any observations which he might have been inclined to publish in his own name for the theses of one of his most distinguished pupils of that year; and having put him in possession of the various facts which he may wish to communicate, leaves him to work up the literature of the subject, and to give a digest of whatever may have been known about it. It is, we presume, in great measure owing to this excellent custom that the continental theses are so very superior to the generality of those coming from Edinburgh; although we are far from wishing to deny that occasionally some of considerable talent emanate from our respected alma mater, and we have no doubt that, with a similar degree of encouragement, they would rise to equal or even greater merit, and add fresh laurels to her well-earned fame.

The name of the author in the present instance is a sufficient voucher for talent and respectability to claim our favorable attention. He comes of a family long distinguished in the profession, more especially in the obstetric part of it; and we can say with pleasure that he has not shown himself unworthy of his distinguished predecessors.

M. Saxtorph enumerates in his first page the various morbid conditions and mechanical causes by which the functions of the cord may be interfered with or injured, and which, from his extensive researches, amounts to a larger number than those who have not paid much attention to the subject would suppose. Its circulation may be impeded by coils round the different parts of the child, by actual knots, by contortions, or by rupture, or by organic changes—as dropsy of the cord, varices, aneurisms, or stricture; it may be filled with hydatids or surrounded by indurated gelatinous matter.

In order to ascertain the relative frequency of prolapsus of the cord our author has endeavoured to collect, from a variety of sources, a very considerable number of these cases, and the result of his inquiries is that, out of a grand total of 116,277 births, prolapsus of the cord occurred 480 times, or in the proportion of 1 in 242. This is not very far from the result of a similar inquiry by Schuré, who found that, in 60,148 cases collected at different hospitals, the cord had prolapsed in 226, or in the proportion 1 in 226.

M. Saxtorph has very properly discountenanced the opinion that prolapsus of the cord is more liable to occur with some positions of the child than with others, and has shown that it may occur with any. He has put the results of his inquiries into a tabular form, which we subjoin not merely in proof of this fact, but because of its value as a statistical reference;

| | Prolapsus of the cord. | Head Presentation. | Presentation of the head and extremities | Nates presentation. | Feet presentation. | Oblique presentation (arm or shoulder.) | |
|---|---|---|---|---|---|---|---|
| Michaelis . | 27 | 19 | 3 | | 5 | | |
| Me. Boivin . | 38 | 32 | 6 | | | | |
| Mauriceau . | 39 | 17 | 5 | | 5 | 10 | 2 abor- |
| De la Motte . | 14 | 10 | 4 | | | | tions. |
| Me. Lachapelle | 36 | 23 | 9 | 1 | 1 | 2 | |
| Me. Lachapelle | 41 | 32 | 7 | 2 | | | |
| Dr. Collins . | 97 | 79 | 7 | 2 | 9 | | |

The author has pointed out the action of the inferior segment of the uterus in surrounding the presenting part of the child so closely as to prevent the funis, under ordinary circumstances, from prolapsing; hence we can easily understand why prolapsus of the cord should be chiefly seen where the uterus has been distended with a large accumulation of liquor amnii, and whereby it has been rendered so spherical that the inferior segment could have no effect in preventing the cord from slipping down between itself and the presenting part. He refers to Professor Michaelis's valuable paper on this subject, of which we have ourselves given an abstract in the First Volume of this Review, (p. 588,) and to which we must refer our readers. The other causes which have been assigned, viz. contracted pelvis, malposition of the child, retort-shaped uterus, with pendulous abdomen, are all, in our opinion, of very doubtful effect; and, like most other authors of the present day, the author omits to mention the child's death as a cause; a circumstance which decidedly has a considerable effect in determining the prolapsus of the cord, and upon which, we suspect, it mostly depends where it occurs in conjunction with a contracted pelvis or an arm or shoulder presentation. Still less can we suppose that the cord being entangled round different parts of the child could act as a predisposing cause, as Mauriceau appears to have stated, and also, more recently, Deneux; we must, however, do our author the justice to say that he merely mentions these as the opinions of different authors, adhering to none in particular himself.

He has devoted considerable attention to the interesting fact which Professor Naegele, jun. has lately brought again under the notice of the profession, viz. that where the placenta is inserted near to the os uteri and the cord rises from its lower edge, prolapsus is very likely to occur:

"Levret (1751) pointed out the fact that the lower the placenta was situated towards the os uteri, the nearer the cord was inserted into the edge of it, and generally at that part where it was in closest proximity to its edge. Zeller, in 1806, showed that the vicinity of the placenta to the os uteri might be an occasional cause of prolapsus of the cord. We also find several cases recorded in Stein of prolapsed cord, where the placenta was situated in the inferior segment of the uterus, with the cord inserted into its lower margin. Deneux has collected some cases from Mauriceau and Smellie where prolapsus of the cord has been accompanied with attachment of the placenta near the os uteri. Dr. Haase relates a case of prolapsed cord in which the placenta was attached to the edge of the os uteri; the cord was returned with difficulty, coming down continually; it was sixteen inches long, and partly inserted into the membranes. Busch has observed many cases in which the prolapsus of the cord was apparently owing to its length and insertion into the edge of the placenta, in two of which it was attached near the os uteri. Dr. Churchill also mentions that in prolapsus of the cord he has repeatedly found the placenta attached near the os uteri, in some of which the cord has been inserted into the edge of the placenta, and which he considers as one of the causes of prolapsus. Carriere d'Azerailles, who is of opinion that the bruit de soufflet is a certain indication of the spot where the placenta is attached, mentions that in twenty-six cases where this sound was heard at the lower part of the uterus, the insertion of the cord was not central, and in some cases where the prolapsus of the cord had required either turning or the forceps he affirms that when he introduced his hand along the cord up to its insertion in the placenta he not only found this quite at the edge, but such that when the membranes gave way the cord unavoidably prolapsed. Still, as we have before observed, none of these authors looked upon this circumstance as one of the chief causes of prolapsus of the cord. F. H. Naegele was the first who paid particular attention to this subject and to its prognosis and treatment. He has described five cases of prolapsed cord accompanied with insertion of the placenta near the os uteri: the cord in every instance was inserted into the edge of the placenta and invariably on the side which was nearest to the os uteri, and in two cases it was even inserted into the membranes, so that its vessels for the space of an inch were separate before they reached the edge of the placenta. He has also communicated an examination of fifty placentæ, all of which he found attached to the lower and lateral portions of the uterus, in two only of which he found a central insertion of the cord, in the rest its distance from the edge varied from an inch and a quarter to an inch and a half; in eleven cases the cord was inserted near the upper edge of the placenta, in all the others near the lower edge. He therefore considers that the insertion of the cord near the orifice of the uterus must decidedly be looked upon as an important predisposing cause of prolapsus, and especially so the nearer the cord is inserted into that edge of the placenta which is nearest to the os uteri. If the edge of the placenta reaches to the os uteri and the insertion of the cord is at this spot, or still more if the umbilical vessels are distributed upon the membranes at a little distance from the edge of the placenta, presentation of the cord becomes a necessary result, so that when the membranes give way prolapsus becomes unavoidable." (p. 34.)

This coincidence of circumstance is doubtless very curious, and in a practical point of view well worthy of attention; we can quite imagine that the insertion of the cord into the lower edge of the placenta which is bordering closely upon the os uteri, would render its prolapse almost unavoidable; but our own numerous cases of partial presentation of the placenta where the insertion of the cord has *not* been marginal convince us that this circumstance is not so invariable as Professor Naegele, jun. would suppose. If it were we ought to meet with prolapsus of the cord in the majority of cases where the placenta was partially presenting: but experience proves that it is not so.

M. Saxtorph, in pointing out the mortality of children where the cord presents, which is so large that out of 356 births only 161 children were born alive, endeavours to investigate the different sources of danger to the fœtus, in doing which he brings much interesting matter to bear upon his subject. He shows that the degree of cold to which the cord may be exposed when prolapsed will rarely act injuriously upon the child's life, and that it is the pressure upon the cord during labour which is the cause of death. Hence it is that the membranes remaining unruptured until an advanced period of labour is so important in ensuring the birth of a living child in these cases. The author gives an excellent summary on this point, which is worthy of notice:

"So long as the membranes have not given way experience proves that the cord presenting is but little affected by pressure, so that it still retains a strong and firm pulsation. We have shown that so long as the membranes are unruptured the cord which presents may change its position, and thus avoid compression. If the membranes remain whole whilst the presenting part descends into the cavity of the pelvis, and if the early stages of labour are retarded, it generally happens that the last stage, during which the prolapsed cord is particularly exposed, passes over very quickly, which is of the greatest importance in preserving the life of the child, if we have left the case to be completed by the natural effort. Moreover, if we consider that hastening of the labour by artificial extraction is indicated, nothing can be a greater assistance to the operation than the membranes being unruptured. If the child is to be delivered footling, the head still remaining moveable at the upper aperture of the pelvis, the membranes being unruptured afford greater mobility of parts. Lastly, if we think it right to wait whilst the head is pressed deeply into the pelvic cavity in order that it may be extracted by the forceps, it will be less dangerous to the life of the child to wait the time which is necessary for the operation whilst the membranes are still whole." (Part ii. p. 14.)

Although a firm, turgid, strongly pulsating cord be an undeniable proof of the child's life, experience has shown us that we are not to come too hastily to the opposite conclusion, viz. of the child being weakly or dead because the cord is thin, soft, and without pulsation; for cases every now and then occur which show that, even with this state of the cord, life was not extinct. The entire extinction of pulsation in the cord, which is usually looked upon as a certain proof of the child's death, is a symptom which must be taken with some degree of caution; for cases have occurred which show that even here we are not justified in giving up hope. The instance which Dr. Evory Kennedy has related proves this very strongly. M. Saxtorph has examined this important point very thoroughly, and collected some very interesting evidence upon the subject.

"We find in numerous authors so many cases recorded of a child being born alive where the cord had been some time prolapsed without any pulsation that there is every reason to suppose that life is not always extinguished simultaneously with sensible pulsation, nor ought we to suppose that the safety of the fœtus is so dependent on the interval which has elapsed since the last pulsation as to feel justified in desisting from every attempt to preserve the child because we can detect no pulsation in the prolapsed cord. We will quote a few instances where the life of the child had been preserved by the aid of art, although the cord had ceased to beat for a considerable time. Thus, Hueter has given a case of a woman in her third labour, and where the midwife had sent for him on account of the cord being prolapsed. He found the head in the first vertex position, and close by the lambdoidal suture a considerable coil of a slender cord without any pulsation, all motion of the child had ceased, the liquor amnii came away putrid and mixed with a large quantity of meconium. Since it was therefore so doubtful that the child's life could be saved he thought it needless to attempt artificial extraction, and merely returned the coil. As the pains increased a living child covered with meconium was born in a few hours by natural efforts. Busch has given the case of a primipara, aged thirty-two, who was brought into the Lying-in Hospital from a distant part of the city in a winter's morning, when the thermometer was at 14° Fahrenheit. She had been sent thither by the midwife, after having had pains through the whole night; towards morning the membranes had burst, and a large coil of cord had prolapsed externally. He found a coil of cord, more than six inches in length, hanging from the external parts, cold and without pulsation; the child was presenting with the nates in the first position, and the os uteri nearly dilated. Having put the cord into warm water he was after a while enabled to perceive weak pulsations, and therefore turned the child, brought down the feet and delivered the head afterwards with the forceps. The child, although born in a state of asphyxia, recovered in half an hour. A similar case occurred a few years ago at our own lying-in hospital, which Professor Eschricht kindly communicated to me. A woman in whom labour had already commenced and where a coil of cord was hanging from the external parts, came to the hospital on foot during a very severe winter's night, from a distant part of the town, called Christianhoevn. On being admitted the loop of cord, which was cold and without pulsation, was enveloped in cloths wrung out of hot water. A feeble pulsation was restored, the child was turned and delivered alive." (Part ii. p. 18.)

It has been shown that the position of the cord in the pelvis is very far from being immaterial, as regards the danger to the child's life. The same rule that has long since been taught by Naegele, viz. that in all footling births, whether having originally presented with the nates or feet, or the feet having been artificially brought down in the operation of turning, it is very desirable to direct the cord towards one of the sacro-iliac synchondroses, as being more safe from pressure in this situation; so in presentations or prolapsus of the cord experience shows that the prognosis will be a good deal influenced by the position of the cord. Boer considered that the anterior part of the pelvis is the most dangerous for the cord. In the elaborate report which Professor Busch published of the cases at the Berlin Lying-in Hospital, occurring between 1829 and 1835, of thirty-nine cases of prolapsed cord he found that the child's life was more endangered when the cord was in the anterior parts of the pelvis, even before the membranes were ruptured, where the pains were strong.

M. Saxtorph has also investigated very carefully the manner in which death is produced by pressure upon the cord; and having studied most of the pathological investigations upon the subject, comes to the conclusion that it is identical with asphyxia. A variety of opinions have been broached on this point, some considering that death was produced by anemia and syncope, others by plethora and apoplexy, and others by suffocation and asphyxia. The appearances after death appear to justify the conclusion that it is the latter cause:

"On external inspection most observers have found the surface livid, occasionally, nevertheless, pale; most frequently marked with distinct livid spots. (*Zeller, Capuron.*) The nails, tips of the fingers, and face are livid; this, however, varies, but still the discoloration is pretty uniform in the lips. The globes of the eyes and tongue are swollen, (*Mende:*) this latter is seldom protruded beyond the mouth. The cavities of the mouth and nose contain a bloody mucus, and sometimes liquor amnii mixed with meconium, (*Dubois;*) the face is usually placid, as of an infant asleep; the extremities, especially the fingers and toes, spasmodically contracted. (*Schmitt.*) The epigastric region is but little expanded (*Wigand*), and the external surface of the body is often smeared with meconium if this has been mixed with the liquor amnii. (*Busch, Dubois.*) In the upper and back part of one or other of the parietal bones, on removing the cranial integuments, a gelatinous fluid, sometimes mixed with extravasated blood, but rarely pure blood, is found. The cranial bones are as injected with red blood as if it had been done artificially. The venous sinuses, the vessels of the dura and pia mater, and of the brain, are so gorged with blood, that innumerable minute drops of blood exude by the slightest pressure on the brain. The above extravasated, bloody, or gelatinous fluid is rarely found at the base of the brain, on its surface, or in the ventricles. This appearance has induced many authors to consider that apoplexy has been the cause of death...... In the thoracic cavity, if the child has not breathed and no attempts have been made to inflate the lungs, we shall find the pericardium covering the lungs, and containing but a small quantity of serous fluid. The heart distended with black blood, seldom coagulated into a polypoid form. The coronary and proper vessels of the heart are so gorged that they could not have been more finely filled even by the most successful injection. The vena cava and other large veins are full of blood, but the arteries generally empty.......... The lungs, in the cavities of the pleura, collapsed, like liver in appearance, dense, compressed, of a dark red colour, and sinking in water.... ...In the abdominal cavity *Kohlschwetter* in two instances has found the liver so gorged with black blood as to resemble one large coagulum. This has also been confirmed by many others." (Part ii. p. 34.)

In the treatment of prolapsus of the cord various modes have been adopted by different authorities. 1st. To leave it entirely to nature. 2d. To replace the prolapsed portion and retain it there: and then either to let the labour take its course, or terminate it by art. 3d. To turn the child with or without artificial extraction. 4th. To terminate the labour by the forceps.

We have already referred to our former notice of Professor Michaelis's interesting observations on the reposition of the cord; it is a subject which we again recommend to the attention of practitioners, and feel justified in so doing, from the fact that in twenty-five cases of prolapsus of the cord he has succeeded in saving the lives of twenty-one children by the reposition. The arguments for and against these different modes of treatment are subjects which are sufficiently considered in the modern midwifery, not to require our entering upon them. There is no doubt but that the treatment will vary much according to the circumstances of the individual case, and that in many instances the forceps proves of great value either in delivering the head which presents, or in the latter part of a delivery where the child has been turned. To Professor Busch, of Berlin, are we much indebted for pointing out the value of the forceps in all cases threatening pressure on the cord during the passage of the head, by which means he has rendered turning a far more successful operation, as regards the child's life, than ever it was before.

M. Saxtorph's essay does him much credit; and we feel assured we cannot pass a better compliment upon it than by saying that it is well worthy of his name.

---

